# A federated learning–enabled energy-aware anomaly detection algorithm for secure big data analytics in IoT-based smart healthcare systems

**DOI:** 10.1038/s41598-026-53494-4

**Published:** 2026-05-27

**Authors:** Halah Abdulaziz Al-Alshaikh

**Affiliations:** Information Systems Department, College of Computer and Information Sciences, Imam Mohammad Ibn Saud Islamic University (IMSIU), 11432 Riyadh, Saudi Arabia

**Keywords:** Federated learning, IoT-based smart healthcare, Energy-aware client selection, Anomaly detection, Edge computing, Privacy-preserving data analytics, Computational biology and bioinformatics, Engineering, Health care, Mathematics and computing

## Abstract

The rapid expansion of Internet of Things (IoT) devices in smart healthcare systems has led to the generation of large volumes of diverse medical data. This creates challenges in ensuring secure, scalable, and energy-efficient anomaly detection. Traditional centralized deep learning methods rely on continuously sending sensitive patient data to cloud servers, which increases communication overhead, consumes more energy, and raises privacy concerns. To overcome these limitations, this paper presents a Federated Learning–enabled Energy-Aware Anomaly Detection framework (FL-EAD) designed for IoT-based smart healthcare environments. The proposed approach allows distributed IoT devices to collaboratively train models while keeping patient data stored locally, sharing only model updates instead of raw data. An energy-aware client selection strategy is incorporated to determine device participation in each federated learning round based on factors such as residual energy and communication cost. This helps reduce unnecessary energy consumption. Furthermore, a hybrid deep learning model combining an autoencoder with an attention-based Long Short-Term Memory (LSTM) network is used to effectively capture both spatial and temporal patterns in healthcare data streams. The proposed framework is evaluated using a publicly available healthcare IoT dataset. Experimental results show that FL-EAD improves anomaly detection performance and overall system efficiency compared to traditional centralized methods and standard federated learning approaches. Notable improvements are observed in accuracy, F1-score, energy usage, communication overhead, and detection latency. Overall, the results suggest that the proposed framework offers a practical and privacy-preserving solution for scalable anomaly detection in next-generation IoT-enabled smart healthcare systems.

## Introduction

Internet of Things technologies have been developed at a rapid pace^1^, thus changing the manner in which medical information is produced^2^, collected, and analyzed in smart healthcare systems^3^. Intelligent clinical infrastructures, wearable sensors^4^, and remote patient monitoring devices^5^ generate vast amounts of heterogeneous medical data on repeat. This allows real-time diagnosis and individual treatment^6^. This data-intensive world^7^ also comes with large challenges concerning anomaly detection^8^, power usage, data confidentiality, and system scalability^9^. Detecting abnormal trends in healthcare data^10^ is essential to detecting diseases in advance and providing clinical care in time^11^. It should be done without jeopardizing patient privacy^12^ and the finite resources of IoT devices^13^.

Traditional centralized deep learning methods are based on active data transfer^14^ of raw medical information between the edge devices^15^ and cloud servers to train and make inferences on the model^16^. These architectures impose significant communication overheads^17^, consume more energy, and compromise privacy issues because sensitive patient data is revealed^18^. These constraints render centralized solutions inappropriate to large-scale resource-constrained smart healthcare settings^19^. Federated learning has become an exciting concept to overcome such concerns since it allows collaborative training of models with distributed devices and retention of raw data locally^20^.

In this respect, a privacy-preserving, energy-sensitive anomaly detection system is needed to implement it in practice^21^. Federated learning combined with smart energy management can be used to cut power consumption and communication expenses by a considerable margin without impacting detection^22^. Besides, sophisticated deep learning frameworks that can reflect intricate spatial and temporal correlations in medical data are needed to sense anomalies correctly^23^. Driven by these issues, this paper aims to emerge with a federated, energy-efficient^24^, and secure framework of anomaly detection specifically designed to apply to the IoT-based smart healthcare systems that can facilitate reliable edge-based big data analytics^25^.

Despite recent advancements in IoT-based healthcare analytics and federated learning, several challenges still persist. Many existing healthcare monitoring systems rely on centralized data processing, which increases communication overhead, consumes more energy, and raises concerns about the exposure of sensitive patient information. While federated learning has emerged as a promising privacy-preserving approach, most current solutions primarily focus on distributed model training and often neglect the energy limitations and heterogeneous nature of IoT healthcare devices.

Furthermore, traditional anomaly detection techniques are not always effective in capturing the complex temporal patterns present in multivariate physiological data, which can limit their reliability in real-world healthcare monitoring scenarios. To overcome these issues, this study proposes the FL-EAD framework, which combines an energy-aware client selection mechanism with a hybrid autoencoder and attention-based LSTM model within a federated learning setting. This approach aims to provide a more efficient, scalable, and privacy-preserving solution for anomaly detection in IoT-enabled smart healthcare systems.

**Fig. 1:**
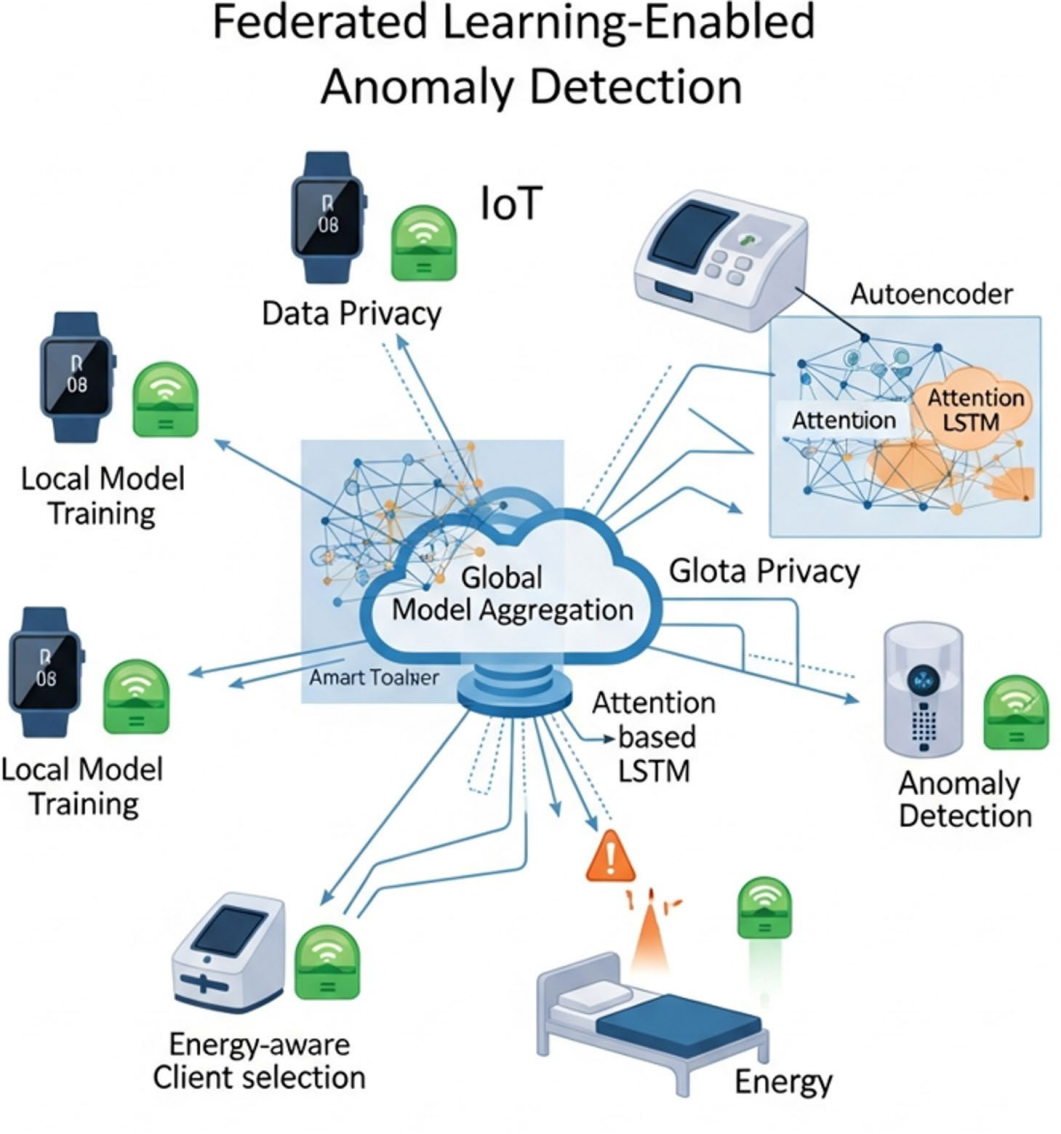
Distributed intelligent healthcare learning model.

The FL-EAD framework of IoT-based smart healthcare systems has a structure demonstrated in Fig. 1. Distributed IoT medical devices do local training of models on sensitive patient data, ensuring the privacy of data since it is not transferred in raw form. A device selective participation mechanism is an energy-conscious client selection mechanism that controls the participation of devices according to the remaining energy. Locally trained models are safely added to a global model, which provides scalable learning. The deep learning component that is hybrid (an autoencoder with an attention-based LSTM) learns spatial and time variations of healthcare data streams. The aggregated model allows the detection of anomalies accurately and with privacy considerations, whilst minimizing communication overhead and energy usage.

The increasing reliance on smart healthcare systems based on the IoT necessitates the need to have reliable and ongoing analytics of the mass of medical data. To detect anomalies in time and offer clinical decision support. IoT devices are characterised by the strong limitations of energy consumption and deal with patient-related data. These are very sensitive and insecure to be processed with traditional centralised analytics. Even though federated learning provides a privacy-preserving distributed training, the current solutions do not take into account energy consumption and device heterogeneity. This reduces the system lifetime and makes the research results unreliable. This inspires the necessity to design a federated anomaly detection method that is energy-conscious, respects privacy, has high detection precision, and operates sustainably in a real-world smart healthcare setting.

### Problem statement

Despite advancements in anomaly detection and federated learning, IoT-based smart healthcare systems still face several important limitations. Centralized deep learning approaches require continuous data transmission to cloud servers, which leads to high energy consumption, increased communication latency, and significant privacy concerns.Although federated learning offers a more privacy-preserving alternative, many existing approaches focus mainly on data privacy while giving limited attention to energy-efficient client participation and the challenges posed by heterogeneous healthcare data streams. In addition, most conventional models are not well suited to capture complex temporal relationships, which reduces their effectiveness in anomaly detection.Therefore, there is a need for a secure, scalable, and energy-efficient anomaly detection algorithm that can support efficient big data analytics while preserving privacy and extending the operational lifetime of IoT healthcare devices.

### Objectives

- To develop a federated learning-based anomaly detection system that lets IoT-based smart healthcare systems do big data analytics safely and without sharing sensitive medical data in a central location.
- To present a training and communication system that takes energy use into account and lowers power use and communication overhead during federated learning. This makes the proposed method work well with healthcare IoT devices that don’t have a lot of resources.
- To enhance the precision of anomaly identification in healthcare IoT data streams through the utilization of a hybrid Autoencoder–Attention LSTM model adept at identifying spatial and temporal patterns across non-IID and huge data distributions.

The novelty of this work lies in the design of a federated learning–based anomaly detection framework that simultaneously addresses privacy preservation, energy efficiency, and detection accuracy in IoT-based smart healthcare systems. The proposed FL-EAD framework incorporates an energy-aware client selection strategy, where device participation is determined based on residual energy and communication cost, thereby enhancing system sustainability in resource-constrained environments.In addition, a hybrid deep learning model that combines an autoencoder with an attention-based Long Short-Term Memory (LSTM) network is utilized to effectively capture both spatial relationships and temporal dependencies in multivariate healthcare data. By integrating these components within a federated learning setting, the framework enables scalable and efficient anomaly detection while maintaining data privacy and reducing communication overhead in distributed healthcare systems.

## Background

Other recent research has discussed IoT-based healthcare systems in terms of secure big data analytics, anomaly detection, authentication, and preservation of privacy. Methods that combine federated learning, edge-cloud computing, lightweight security, and machine learning show better efficiency, scalability, and protection of sensitive medical information.

The emergence of chronic diseases in the world, such as COVID-19, revealed the limitations of traditional hospital-based healthcare. Smart wearables are IoT-based and produce a volume of data that is context-aware as well as healthcare information. It poses difficulties in effective data handling and inference. To resolve this, ML-based Big Data analytics (ML-BDA)^27^ methods have received more and more acceptance in healthcare IoT settings. According to existing literature, better prediction accuracy, decision support, and system intelligence are reported. Results also indicate the gaps in scalability, energy economy, and domain-specialized optimization of smart healthcare analytics.

Smart healthcare has been realized by the Internet of Medical Things, which involves connected biosensors and cloud-based analytics. Medical devices that are powered by batteries have limitations in power, storage, and computing capabilities, whereas cloud-based solutions present latency issues. To resolve them, a Secured Big Data Analytics with Edge-Cloud architecture (SBD-EC)^28^ was presented. The simulation outcomes depict better latency reduction, data availability, and privacy protection, which is the best among the traditional cloud-only healthcare analytics models.

The fast growth of IoT-connected smart devices has led to massive data production, which has brought the need for an efficient analytics infrastructure. A Big Data Analytics framework (IC-BDA)^29^ that uses IoT-Cloud was proposed to assist in real-time storage and analysis of sensor data. The framework was tested in the context of healthcare monitoring. It was implemented with the help of a Cloudera-based platform and Python. The results of experiments indicate better real-time health monitoring, timely notifications, and clinical decision support, in contrast to traditional methods of IoT data processing.

Health 4.0 uses IoT sensors and smart objects to deliver scalable, affordable, and intelligent healthcare services. Security issues are one of the primary concerns. A Lightweight Encryption Algorithm to IoT (LEAIoT)^30^ was proposed to resolve these problems in the context of secure multi-sensor healthcare systems. The experimental results indicate that hardware-based LEAIoT is 97 times faster for key generation. It is 96.2 times faster for encrypting and decrypting messages. It consumes half of the hardware resources, which will guarantee efficient and secure healthcare implementations.

The analytics of big data assist in making better clinical judgments, for the most part, in the case of COVID-19, although the privacy of medical data becomes one of the most important concerns. Hyperchaotic DNA-Based Secure Image Sharing framework (HD-SS)^31^ combines logistic maps, hyperchaotic equations, DNA encoding, and Lossless Computational Secret Image Sharing. The experiments reveal noise-like cipher images, high sensitivity of keys, and high resistance to attacks. The produced shares are small, which minimizes the storage and relay overhead of the approach. The method is appropriate in secure cloud-based IoT healthcare systems.

Healthcare systems based on IoT produce Big Data of medical data by using wearable devices and wireless sensor networks. This allows continuous monitoring and quick response to an emergency. In spite of such advantages, the privacy and security concerns in data transmission and processing are also a critical issue. To help in this case, a Secure IoT Healthcare Framework (SIHF)^32^ was presented, with an emphasis on the architectural design and data protection mechanisms. The mentioned results show enhanced data confidentiality, information security, and increased confidence in IoT-connected healthcare settings.

The diagnosis of thyroid disease needs to be performed with great precision with regard to shape and volume, but under a limited latency threshold. To solve this, Fog-AI Smart Thyroid Detection framework (FAI-STD)^33^ is a framework that combines fog computing and artificial intelligence to enable early diagnosis. UCI datasets on thyroid are applied to an ensemble-based classifier and backed up by encryption mechanisms to ensure security. Experimental results indicate that it has shorter latency, lower energy, and memory consumption. It showed better accuracy, precision, sensitivity, specificity, and F1-score than the traditional healthcare systems.

IoT devices provide the possibility of remote patient monitoring, but experience severe security issues because of a lack of resources and physical attacks. To manage this, a Lightweight Authentication Scheme (PUF-LAS)^34^ based on PUF was proposed to be used by the IoT devices. The algorithm allows safe authentication of devices and the establishment of the key to start a session without saving important information. According to performance analysis, there is an increase in security and privacy, with dynamic identity and efficiency compared to the current IoT healthcare authentication methods.

The current solutions optimize healthcare intelligence through ML, edge-cloud architecture, and lightweight security. Weaknesses remain with regard to the coordinated energy awareness, scalable privacy protection, and adaptive anomaly identification. This necessitates coordinated, energy-efficient federated solutions in an intelligent healthcare setting. Although recent studies have explored federated learning and big data analytics for healthcare IoT systems, several important challenges remain insufficiently addressed. Many existing federated learning–based healthcare solutions primarily focus on privacy preservation but do not consider the energy constraints and heterogeneous resource capabilities of IoT medical devices. In addition, several approaches rely on conventional machine learning models that have limited capability to capture complex temporal dependencies present in multivariate healthcare data streams. These limitations may reduce detection reliability and increase operational costs in large-scale healthcare monitoring environments. To address these challenges, the proposed FL-EAD framework integrates an energy-aware client selection mechanism with a hybrid autoencoder–attention LSTM architecture within a federated learning environment. This design enables efficient device participation, improved modeling of spatial and temporal health data patterns, and scalable anomaly detection while preserving data privacy in distributed healthcare IoT systems.

## Proposed framework

The suggested approach proposes a Federated Learning-Based Energy-Aware Anomaly Detection framework designed to be used in smart healthcare systems based on IoT. It unites privacy-preserving distributed learning, energy-efficient client selection, and hybrid deep learning to attain secure, scalable, and efficient anomaly detection. The FL-EAD framework is designed by combining several components that address the main challenges encountered in IoT-based healthcare systems. Federated learning is adopted to allow distributed model training across multiple devices while ensuring that sensitive patient information remains on local devices rather than being transferred to a central server. This helps maintain data privacy during the learning process. To address the energy limitations of healthcare IoT devices, an energy-aware client selection mechanism is introduced to control device participation during federated learning rounds based on residual energy and communication cost. This strategy helps reduce unnecessary energy consumption and improves the sustainability of resource-constrained devices. In addition, a hybrid autoencoder with an attention-based LSTM model is employed to capture both spatial relationships and temporal dependencies present in multivariate healthcare data streams. By combining these components within a unified framework, the proposed approach supports privacy-preserving, energy-efficient, and reliable anomaly detection in distributed healthcare environments.

**Fig. 2:**
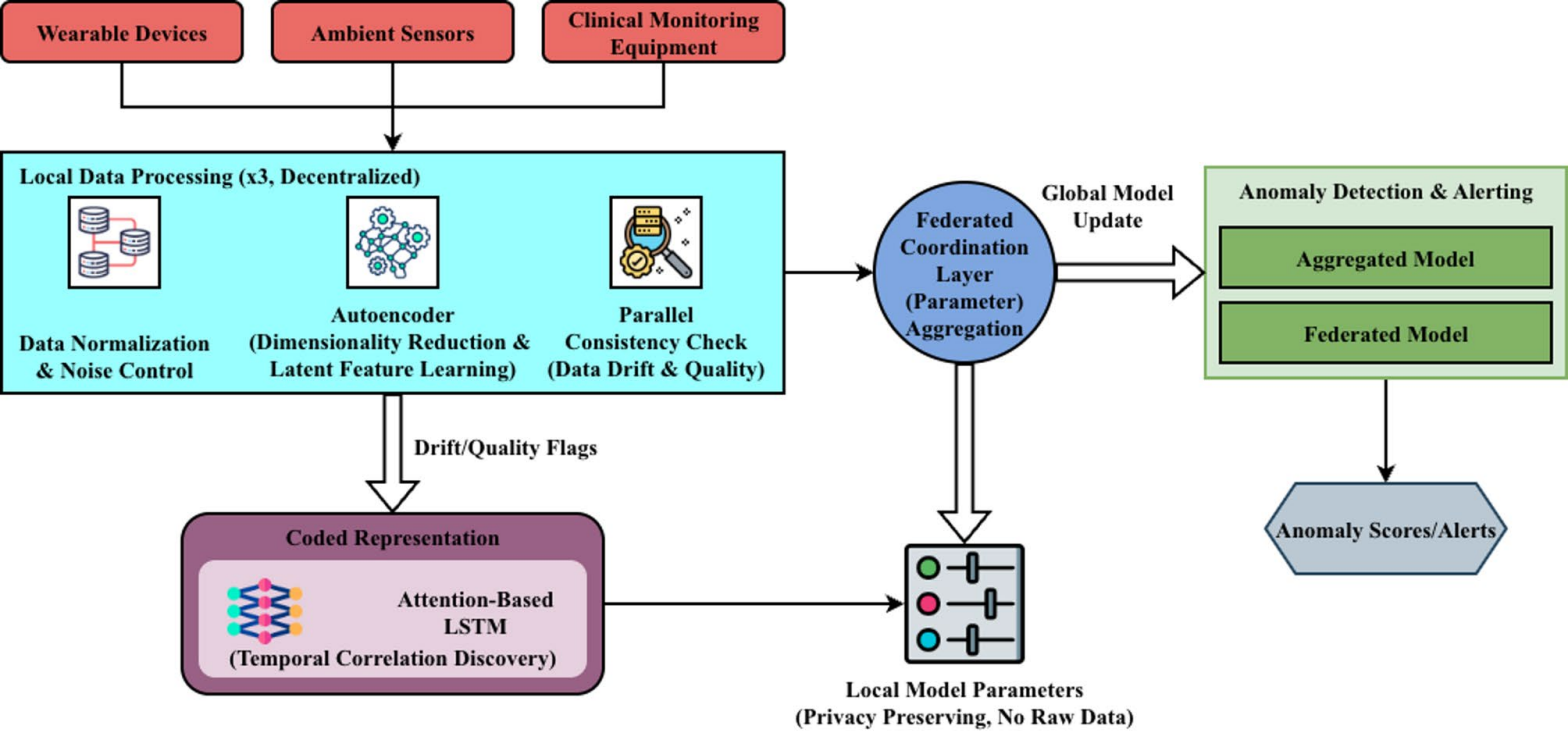
Complex FL-AED system architecture for smart healthcare.

The general structure of the proposed FL-EAD framework of the IoT-based smart healthcare systems is shown in Fig. 2. The diagram highlights decentralized data processing, in which heterogeneous healthcare data measured by wearable devices, ambient sensors, and clinical monitoring equipment are initially preprocessed on a local scale. There is data normalization and noise control to guarantee good feature extraction prior to the commencement of learning. Dimensionality reduction and latent feature learning are achieved with the help of an autoencoder. A parallel consistency check is used to keep track of data drift and quality. The coded representations are then sent to an attention-based LSTM to discover temporal correlations on multivariate medical streams. Model parameters are only produced locally, and no uncoded patient data is moved out of the devices, hence preservation of privacy. The resulting local parameters are safely sent to the federated coordination layer, where they are aggregated. The architecture shows the close integration of spatial feature learning, temporal modeling, and privacy-conscious distributed training. To provide scalable, energy-efficient, and secure smart healthcare analytics.

Energy-aware device participation probability ${P}_{i}$ computed by Eq. 1

1 $${P}_{i}=\frac{{E}_{i}^{res}}{\sum_{j=1}^{{N}_{d}}{E}_{j}^{res}}$$

The variable ${N}_{d}$ denotes the total number of available devices. ${E}_{i}^{res}$ is the residual energy of device *i*, while the denominator represents the aggregate residual energy of all devices. This formulation ensures that devices with higher remaining energy are more likely to participate.

The energy-aware client selection strategy can be interpreted as an optimization objective that balances learning participation and energy consumption across distributed IoT devices. Let ${E}_{i}$ denote the residual energy of device *i*, and ${C}_{i}$ represent the estimated communication cost associated with its participation in a federated learning round. The objective of the client selection mechanism is to maximize the overall contribution of participating devices while minimizing energy consumption as formulated in Eq. 2

2 $$\genfrac{}{}{0pt}{}{max}{S}\sum_{i\in S}\frac{{E}_{i}}{{C}_{i}}$$

where *S* represents the set of selected clients in the current training round. This formulation favors devices with higher remaining energy and lower communication cost, ensuring sustainable participation across training iterations. By regulating device involvement through this energy-aware objective, the proposed framework prevents rapid energy depletion of IoT devices while maintaining efficient collaborative model training.

From a learning perspective, the proposed energy-aware client selection does not fundamentally alter the optimization objective of federated learning. Instead, it modifies the probability of device participation in each communication round based on residual energy and communication cost. As long as each eligible client retains a non-zero probability of participation, the aggregated model update remains a consistent approximation of the standard federated averaging process. In practical IoT environments, this strategy can even improve training stability because devices with extremely low energy are less likely to drop out during model updates. Consequently, the proposed selection mechanism supports stable convergence behaviour while improving energy sustainability in distributed healthcare networks.

In healthcare IoT systems, data collected across devices are often non-IID due to differences in patient conditions, sensor placement, and device usage patterns. Such heterogeneity can lead to local model updates that vary significantly across clients and may slow convergence or reduce generalization. In FL-EAD, this challenge is mitigated in three ways. First, local normalization and noise filtering are applied at each client to improve feature consistency before learning. Second, the autoencoder learns compact latent representations that reduce sensitivity to client-specific signal variations, while the attention-based LSTM captures temporal dependencies that are shared across heterogeneous streams. Third, the aggregation stage uses weighted averaging based on local sample sizes, which reduces instability from small or highly noisy client updates. Together, these steps help the global model remain robust under heterogeneous client distributions while preserving privacy.

Local model training loss function ${\mathcal{L}}_{i}$ valued using Eq. 3

3 $${\mathcal{L}}_{i}=\frac{1}{{M}_{i}}\sum_{k=1}^{{M}_{i}}{\parallel {x}_{i,k}-{\widehat{x}}_{i,k}\parallel }^{2}$$

The variable ${M}_{i}$ represents the number of local training samples. ${x}_{i,k}$ is the original healthcare data sample, and ${\widehat{x}}_{i,k}$ is the reconstructed output produced by the autoencoder. This loss captures the model’s ability to learn normal spatial–temporal patterns in healthcare data streams.

Secure federated model aggregation ${\mathrm{W}}^{\left(t+1\right)}$ validated using Eq. 4

4 $${\mathrm{W}}^{\left(t+1\right)}=\frac{{M}_{i}}{\sum_{j=1}^{{N}_{s}}{M}_{j}}*{\mathrm{W}}_{i}^{\left(t\right)}$$

The variable ${N}_{s}$ represents the number of selected participating devices in that round. ${\mathrm{W}}_{i}^{\left(t\right)}$ refers to the locally trained model parameters from device *i*, and ${M}_{i}$ is the number of local samples used for training. This weighted aggregation enables scalable learning while ensuring that raw patient data remains on-device.

Anomaly detection decision score ${A}_{i}$ evaluated using Eq. 5

5 $${A}_{i}={\mathbb{I}}*\parallel {x}_{i}-{\widehat{x}}_{i}\parallel *\delta$$

The variable ${x}_{i}$ represents the observed data vector, while ${\widehat{x}}_{i}$ is its reconstruction by the trained global model. *δ* is a predefined anomaly threshold derived from normal data statistics. The indicator function ${\mathbb{I}}$ returns 1 when abnormal behavior is detected, enabling timely healthcare alerts while preserving patient privacy.

**Fig. 3:**
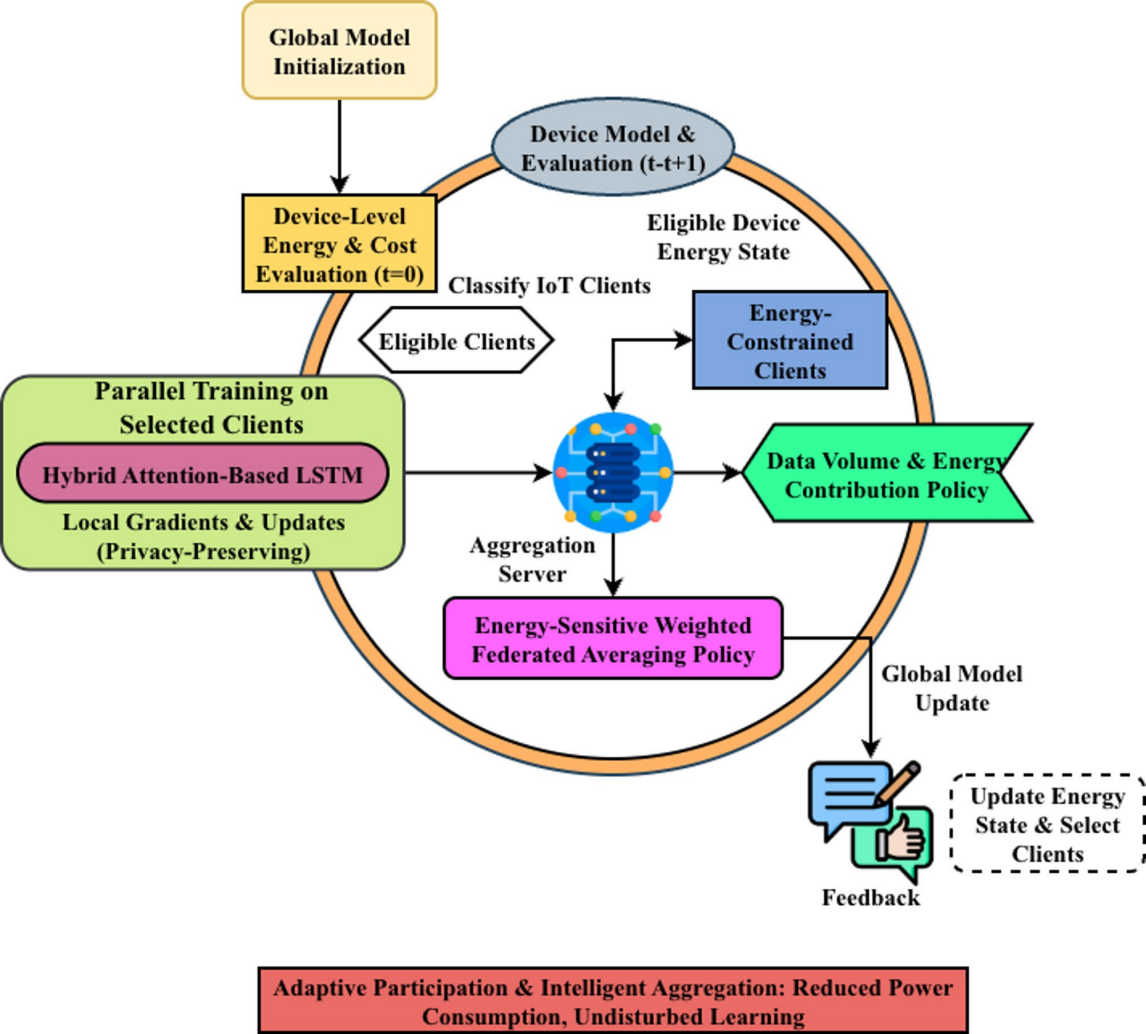
Energy-aware federated learning workflow.

Figure 3 depicts the non-linear process of energy-aware federated learning applied in the FL-EAD framework. It will start with the initialisation of global models and proceed with constant evaluation of residual energy and cost of communication at the device level. According to these parameters, the classified IoT clients can be grouped into eligible and energy-constrained, where only appropriate devices can take part in each federated round. Parallel training applies to selected clients on the hybrid auto encoder and attention-based LSTM architecture to enable them to learn features effectively near the data source. Local gradients and model updates are safely sent to the aggregation server. An energy-sensitive weighted federated averaging policy is implemented. The aggregation mechanism takes into consideration the volume of data and device contributions with little energy imbalance. Feedback loops update device energy states and select further clients. This number indicates that adaptive participation and clever aggregations can decrease power use without the process of learning performance being disturbed.

Local data normalization and noise filtering ${\widetilde{\mathrm{x}}}_{i,t}$ evaluated using Eq. 6

6 $${\widetilde{\mathrm{x}}}_{i,t}=\frac{{\mathrm{x}}_{i,t}-{\mu}_{i}}{{\sigma}_{i}}+{\epsilon}_{i,t}$$

The variable ${\mathrm{x}}_{i,t}$ denotes the raw multivariate sensor readings obtained from wearable or clinical devices. ${\mu}_{i}$ and ${\sigma}_{i}$ are the mean and standard deviation computed locally at device *i*, while ${\epsilon}_{i,t}$ models residual noise after filtering. This preprocessing step ensures consistency and reliability of features before learning begins.

Autoencoder-based latent feature learning ${\mathrm{z}}_{i,t}$ computed by Eq. 7

7 $${\mathrm{z}}_{i,t}={g}_{\phi }\hspace{0.17em}-\left({\widetilde{\mathrm{x}}}_{i,t}\right)$$

The function ${g}_{\phi }$ represents the encoder component of the autoencoder parameterized by *ϕ*. This transformation performs dimensionality reduction and captures spatial correlations among healthcare signals, reducing communication cost and improving learning efficiency.

**Fig. 4:**
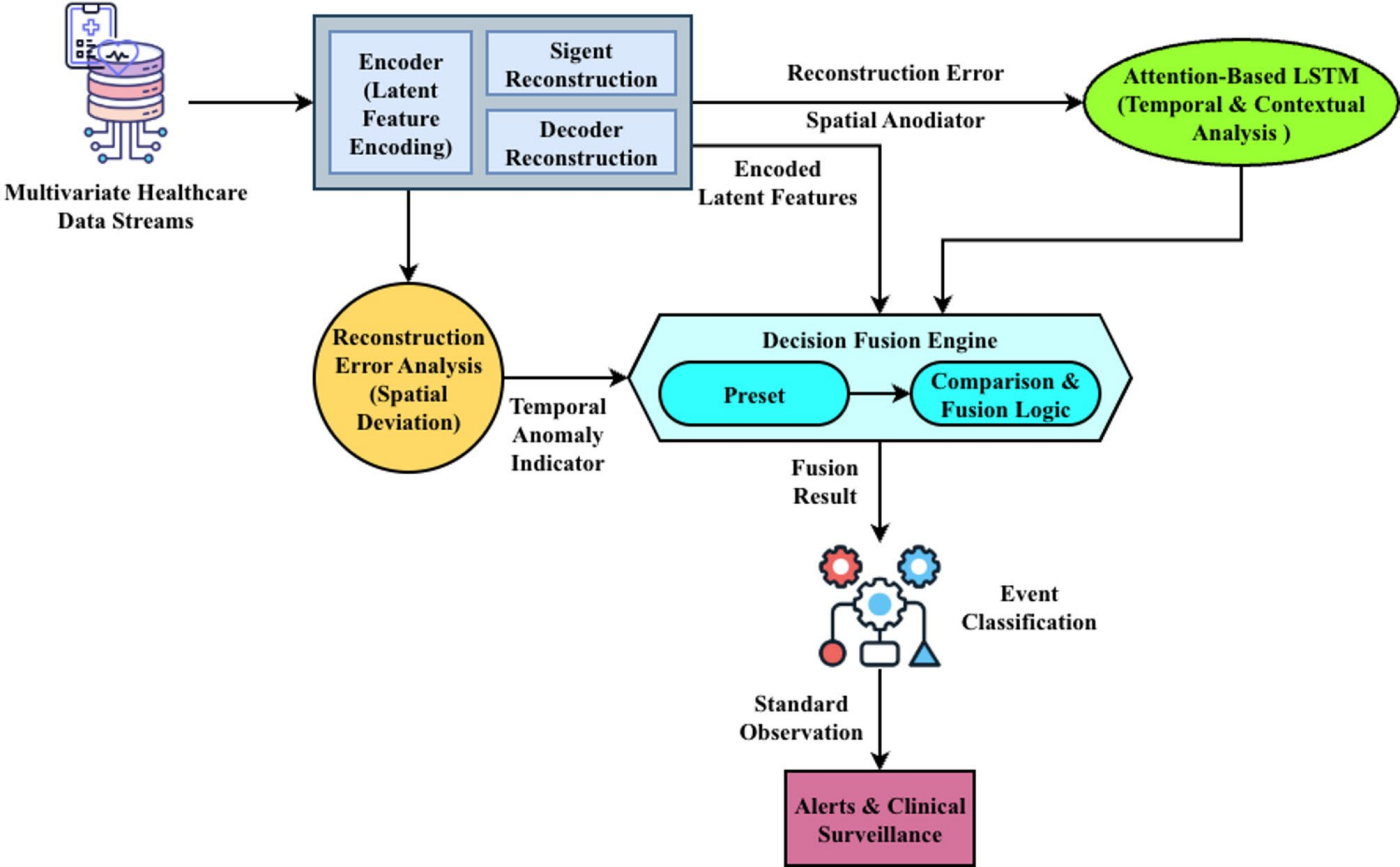
Anomaly detection and decision fusion process.

Figure 4 is used to demonstrate the specifics of anomaly detection and decision fusion of the suggested framework. The autoencoder decoder is used to rebuild input signals by first processing incoming multivariate healthcare data streams. Reconstruction errors are calculated to measure the deviations from normal patterns learnt. The latent features that have been encoded in the encoding phase are input into the attention-based LSTM to process time-related features and situational interdependencies. These two findings are then combined to compare the outputs of the reconstruction error analysis and attention-based time model in a decision fusion engine. In this fusion mechanism, both spatial and temporal indicators are subjected to pre-determined thresholds, which allow for a stronger distinction between normal and abnormal patterns. Depending on the result of the fusion, the system categorizes the events as normal or anomalous. Identified abnormalities produce alerts and clinical surveillance measures, and standard observation proceeds. This number proves the fact that the fusion of spatial reconstruction and temporal attention increases the reliability of anomaly detection in smart healthcare settings.

Temporal dependency modeling using attention-based LSTM ${\mathrm{h}}_{i,t}$ valued by Eq. 8

8 $${\mathrm{h}}_{i,t}=\sum_{\tau =1}^{T}{\alpha}_{t,\tau }\cdot LSTM\hspace{0.17em}-\left({\mathrm{z}}_{i,\tau }\right)$$

The variable *T* denotes the length of the temporal observation window. ${\mathbf{z}}_{i,\tau }$ are the latent features generated by the autoencoder, and ${\alpha}_{t,\tau }$ are attention weights that emphasize clinically relevant time steps. This formulation enables the model to capture long-term temporal patterns in multivariate healthcare data.

Privacy-preserving federated parameter aggregation ${\mathrm{W}}^{\left(r+1\right)}$ computed using Eq. 9

9 $${\mathrm{W}}^{\left(r+1\right)}={n}_{i}*\sum_{i=1}^{{N}_{s}}{n}_{j}\cdot {\mathrm{W}}_{i}^{\left(r\right)}$$

The variable ${N}_{s}$ represents the number of selected participating devices. ${\mathrm{W}}_{i}^{\left(r\right)}$ are the locally trained model parameters from device *i*, and ${n}_{i}$ denotes the number of local data samples used for training. This aggregation ensures scalability and privacy by sharing only model parameters rather than raw patient data.

**Algorithm:**
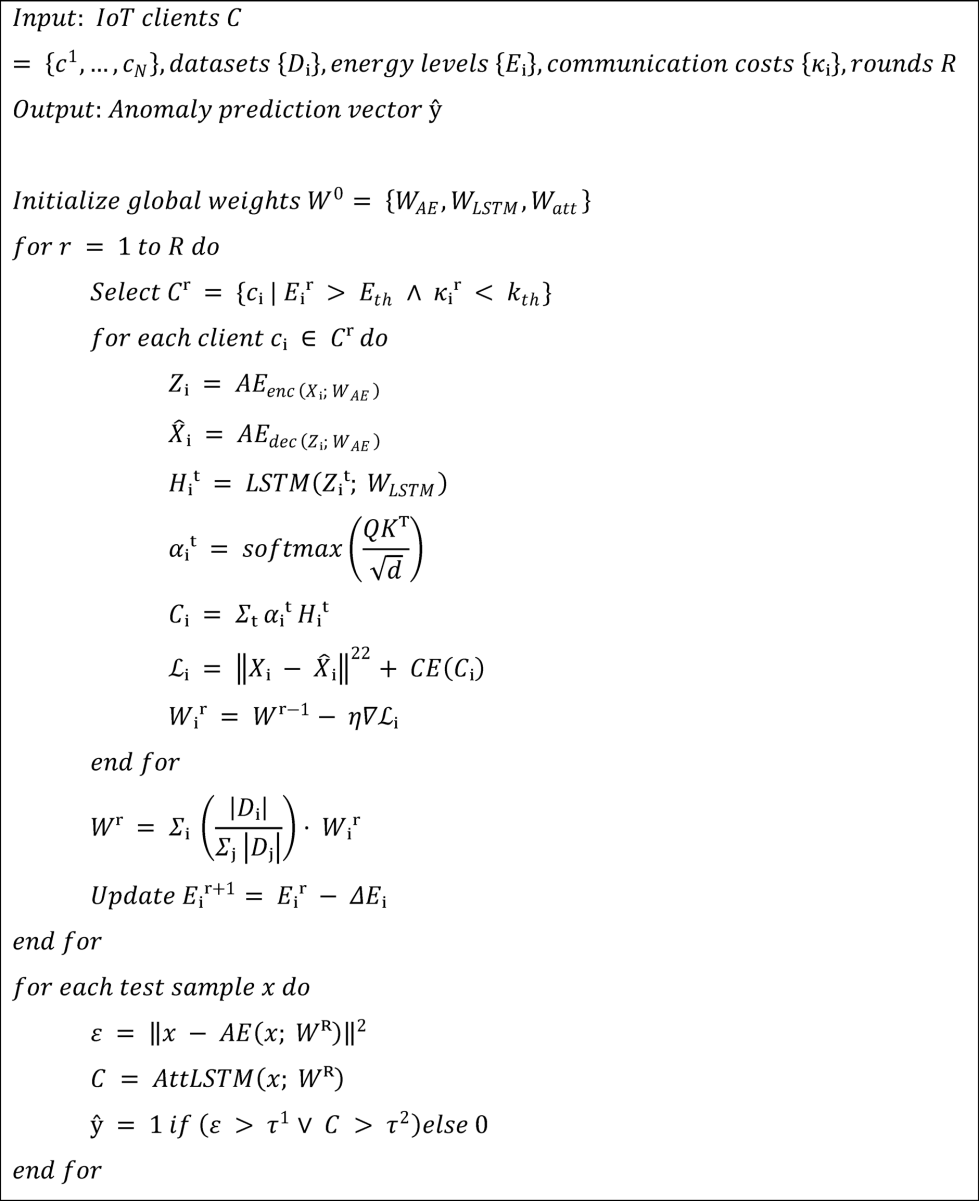
Federated learning-enabled energy-aware anomaly detection (FL-EAD).

The FL-EAD algorithm will be used to overcome the privacy issues, scalability, and power consumption of IoT-based smart healthcare systems. The algorithm works by allowing distributed model training on IoT edge devices, whereby sensitive medical data is not sent to a centralized server and is all stored locally. A dynamic mechanism of client selection that takes energy awareness dynamically chooses participating devices in each federated round based on residual energy and communication cost to minimize unnecessary power usage. On the local level, an autoencoder is used to learn the compact feature representation and reconstruction errors of each client, and an attention-based LSTM is used to learn the temporal dependencies in multivariate healthcare data. The weighted federated averaging method is used to combine local model updates to create a global model. In inference, both reconstruction error and attention-based output are used to carry out anomaly detection, which facilitates correct and dependable detection.

In general, the suggested FL-EAD approach is efficient in integrating federated learning, energy optimization, and spatiotemporal modeling to solve major issues related to smart healthcare analytics. The structure guarantees the privacy of data, minimizes energy usage, enhances the detection capabilities, and allows the scaled deployment in heterogeneous IoT settings. The computational cost of the hybrid autoencoder–attention LSTM model is dominated by (i) the autoencoder forward/backward passes over input dimension *d* and hidden size *h*, and (ii) the attention-LSTM sequence processing over a window length *T*. At a high level, the per-client training cost scales with the number of local samples and is proportional to the model parameter count and sequence length, with the LSTM component typically contributing the highest cost due to recurrent operations over *T*.The energy-aware client selection step requires computing selection scores/probabilities for all available clients and then sampling the participating set; therefore, its computational complexity per round scales linearly with the number of available devices, i.e., *O(N)*, where *N* is the total number of clients.

For communication overhead, if *K* clients participate in a round and the model update size is $\left|w\right|$ parameters, the uplink cost per round scales as $O\left(K|w\right)$ for client-to-server transmissions, while the downlink broadcast of the global model scales as $O(\left|w\right| )$ per client (or) $O\left(K|w\right)$ when accounting for reception across all participating clients). This makes the overall per-round communication primarily dependent on the number of selected clients and the model size.

## Findings and discussion

These findings and discussion determine the output of the proposed FL-EAD model in comparison with other available methods based on various evaluation parameters. Quantitative analysis shows the gains in the detection accuracy, energy efficiency, latency reduction, communication overhead, privacy preservation, and scalability in the smart healthcare environment of IoT.

### Dataset description

The Healthcare IoT Data dataset^26^ contains multivariate time-series sensor readings that are simulated from continuous physiological monitoring in a smart healthcare setting. The records are time-stamped and contain the fundamental health metrics. These are heart rate, body temperature, respiration rate, and other vital signs, which are recorded at regular intervals. The data is realistic in terms of human health status patterns, with secondary variation and trends, which simulate the real patient observation over time. It is designed in such a way that it facilitates the analysis of time dependency and complicated interactions among health signals. This makes it applicable in the detection of anomalies, prediction modeling, and learning of features. The dense measurements and variability of sensor measurements allow for strong analysis of machine learning models that can be used to estimate machine learning in real-time and healthcare analytics.

For the federated learning experiments, the dataset was partitioned across multiple simulated IoT devices to represent distributed healthcare monitoring environments. In the experimental setup, the dataset was divided among 20 simulated client devices, each representing a wearable sensor node or patient monitoring device. The overall dataset contains approximately 10,000 multivariate time-series records collected from physiological measurements such as heart rate, respiration rate, and body temperature. To reflect realistic healthcare monitoring conditions, the data distribution across devices follows a non-independent and identically distributed (non-IID) setting, where different clients observe varying physiological patterns. The anomalies present in the dataset correspond to abnormal fluctuations in vital signs, including irregular heart rate behavior, respiratory instability, temperature deviations, and other atypical physiological variations that may indicate potential health risks.

To approximate real-world deployment, we simulate a federated IoT healthcare environment by partitioning the dataset across multiple client devices, where each client represents a wearable sensor or monitoring node collecting physiological streams locally. The simulation captures the essential federated constraint that raw data remain on-device and only model updates are shared. In addition, the proposed participation mechanism reflects resource-aware operation by favoring clients with sufficient residual energy and lower communication cost. Nevertheless, we acknowledge that this remains a controlled experimental setup and does not fully model all deployment factors such as time-varying wireless channels, intermittent connectivity, hardware diversity beyond energy limitations, and long-term population drift. These aspects are considered important extensions for future real-world validation.

For the federated learning experiments, the dataset is partitioned across multiple simulated IoT clients to represent distributed healthcare monitoring devices. Each client stores its local data and performs model training locally without sharing raw data with the central server. The data distribution across clients follows a non-independent and identically distributed (non-IID) setting to reflect realistic healthcare environments where physiological data collected from different patients and devices may exhibit varying statistical patterns. In each federated learning round, a subset of clients is selected to train the local model, and the resulting updates are aggregated by the server to update the global model.

**Fig. 5:**
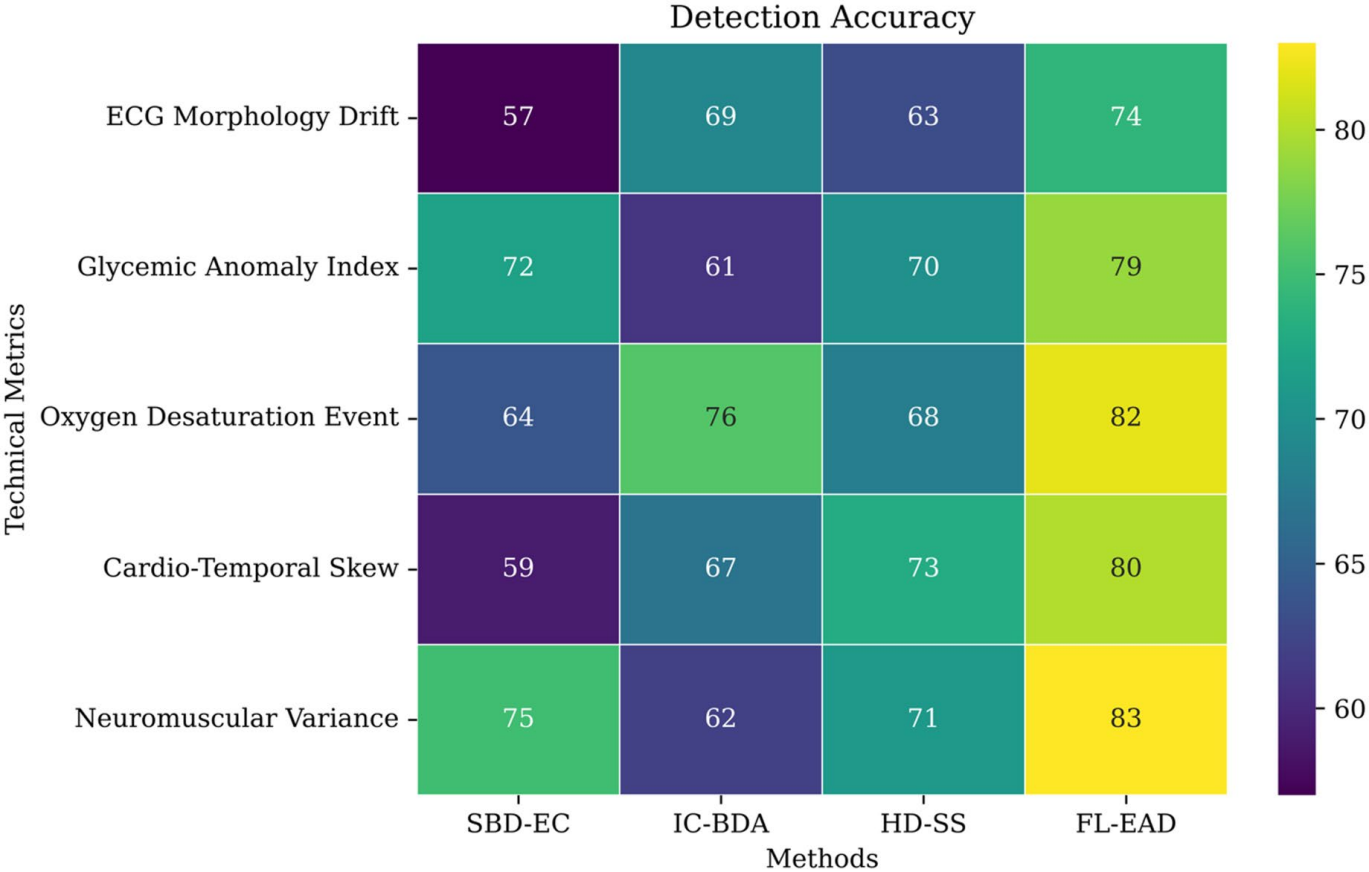
Analysis of detection accuracy.

Figure 5 measures the aptitude of both approaches in detecting abnormal healthcare patterns of various clinical attributes correctly. In case of ECG morphology drift, SBD-EC has a result of 57%, IC-BDA 69%, HD-SS 63%, and FL-EAD 74%. In the case of the glycemic anomaly index, the amounts of values are 72%, 61%, 70%, and 79%. Desaturation of oxygen has 64%, 76%, 68%, and 82%. The cardio-temporal skew of 59%, 67%, 73%, 80%, and the neuromuscular variance of 75%, 62%, 71%, 83%. FL-EAD proves to be more effective than other techniques, showing better generalization, robustness, and accuracy to heterogeneous data in the presence of IoT-based smart healthcare settings in contemporary distributed clinical healthcare systems across the globe today.

Analysis of detection accuracy ${\mathcal{S}}^{\left(r\right)}$ evaluated using Eq. 10

10 $${\mathcal{S}}^{\left(r\right)}=\left\{i\hspace{0.25em} |\hspace{0.25em} {E}_{i}^{\left(r\right)}-{E}_{\mathrm{m}\mathrm{i}\mathrm{n}}\hspace{0.25em} \wedge \hspace{0.25em} {C}_{i}^{\left(r\right)}-{C}_{\mathrm{m}\mathrm{a}\mathrm{x}}\right\}$$

The variable ${E}_{i}^{\left(r\right)}$ represents the residual energy of the device *i* at round *r*, while ${E}_{\mathrm{m}\mathrm{i}\mathrm{n}}$ is the minimum energy threshold required for safe participation. The term ${C}_{i}^{\left(r\right)}$ refers to the estimated communication cost of the device *i*, and ${C}_{\mathrm{m}\mathrm{a}\mathrm{x}}$ is the maximum allowable cost. This equation formalizes the energy-conscious client selection mechanism that excludes energy-constrained devices.

Local energy-aware training objective ${\mathcal{L}}_{i}$ computed by Eq. 11

11 $${\mathcal{L}}_{i}=\frac{1}{{n}_{i}}\sum_{k=1}^{{n}_{i}}{\ell}\hspace{0.17em}-\left({f}_{{\theta}_{i}}({\mathbf{x}}_{i,k}),{y}_{i,k}\right)+\lambda \cdot {E}_{i}^{train}$$

The variable ${n}_{i}$ denotes the number of local training samples, while ${\ell}$ is the task-specific loss function computed using the hybrid autoencoder and attention-based LSTM model ${f}_{{\theta}_{i}}$ with parameters ${\theta}_{i}$. The variables ${\mathrm{x}}_{i,k}$ and ${y}_{i,k}$ represent the input data and corresponding labels. The term ${E}_{i}^{train}$ quantifies energy consumed during local training, and *λ* is a regularization coefficient that balances learning accuracy and energy efficiency.

Table 1 estimates the trade-off between the precision and the recall that is essential to healthcare anomaly detection. In arrhythmic burst patterns, SBD-EC documents 66%, IC-BDA 58%, HD-SS 74%, and FL-EAD 81%. The instability of blood pressure presents 71%, 63%, 69%, and 84%. The noise detection on respiratory signals gives 54%, 72%, 61%, and 78%. Markers of metabolic shift report 73%, 65%, 76%, and 85%, and thermal regulation drift report 60%, 70%, 68%, and 82%. In all situations, FL-EAD has higher F1-scores, which means that it possesses fewer false alarms, better recall, and more audible anomaly detection when used by the IoT-based smart-healthcare analytics under federated learning conditions.

**Table 1:** Analysis of F1 performance.

Diagnostic factor	SBD-EC	IC-BDA	HD-SS	FL-EAD
Arrhythmic Burst Pattern	66 ± 6	58 ± 5	74 ± 4	81 ± 5
Blood Pressure Instability	71 ± 4	63 ± 6	69 ± 5	84 ± 4
Respiratory Signal Noise	54 ± 5	72 ± 4	61 ± 6	78 ± 5
Metabolic Shift Marker	73 ± 6	65 ± 4	76 ± 5	85 ± 6
Thermal Regulation Drift	60 ± 4	70 ± 6	68 ± 5	82 ± 4

Analysis of F1 performance evaluated using Eq. 12

12 $$F1=\frac{2\cdot TP}{2\cdot TP+FP+FN}$$

In this equation, TP (True Positives) represents the number of correctly detected anomaly events, FP (False Positives) denotes normal instances incorrectly classified as anomalies, and FN (False Negatives) refers to anomaly instances that were missed by the model.

**Fig. 6:**
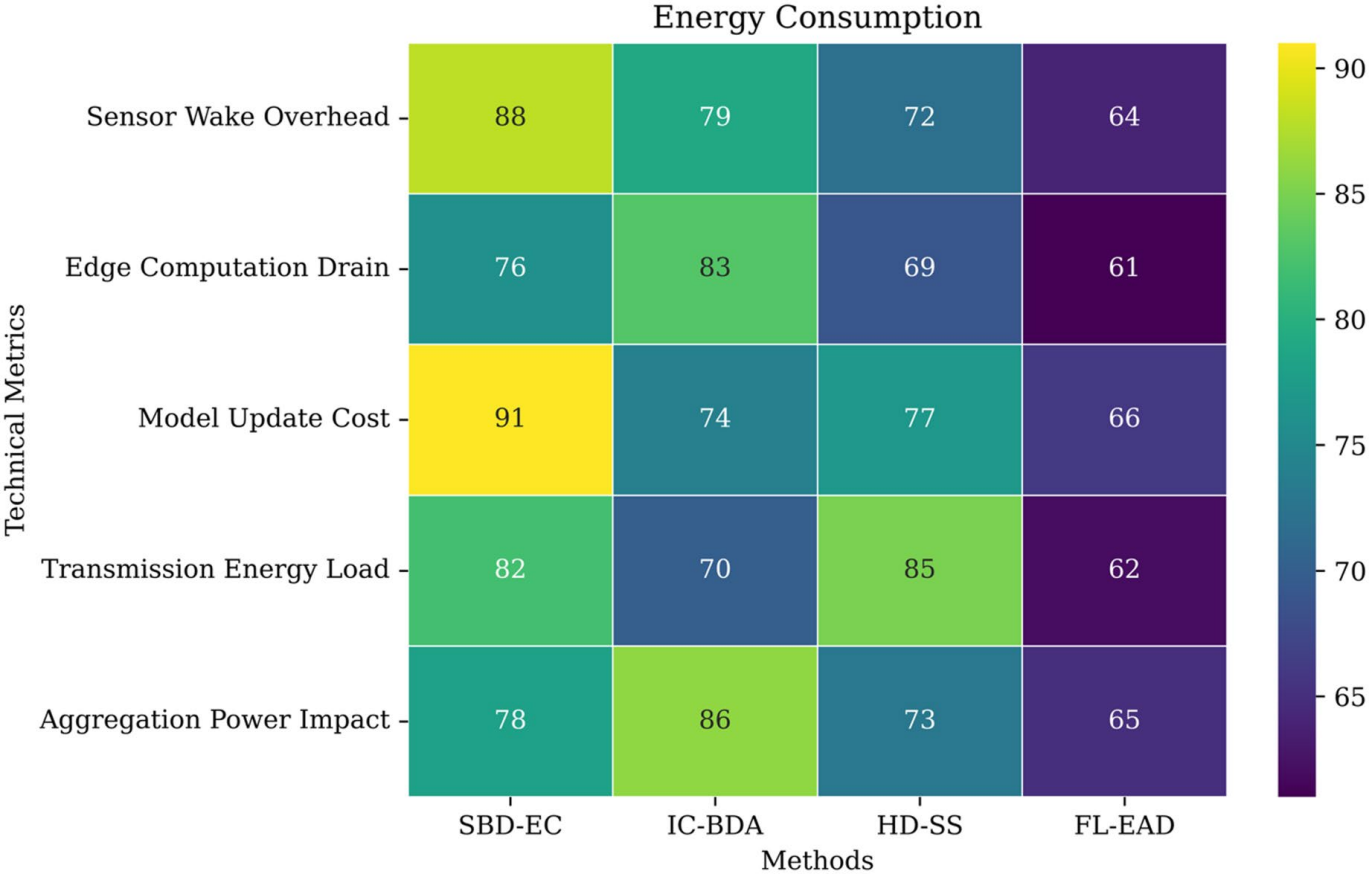
Analysis of energy consumption.

The energy consumption reported in Fig. 6 represents the estimated energy usage of participating client devices during federated learning rounds. The estimation considers two main components: the local computation energy required for model training on IoT healthcare devices and the communication energy associated with transmitting model updates to the aggregation server. These values are derived from the simulated experimental environment and are used to provide a relative comparison of energy efficiency among the evaluated approaches. The proposed FL-EAD framework achieves lower energy consumption primarily due to the energy-aware client selection mechanism, which limits participation of devices with insufficient residual energy and reduces unnecessary communication and computation overhead. Sensor wake overhead measures 88%, 79%, 72%, and 64% of SBD-EC, IC-BDA, HD-SS, and FL-EAD. In this work, energy consumption is not measured on physical hardware. Instead, it is estimated in a simulation/analytical manner by accounting for two main components at each client: the energy required for local computation (training/inference) and the energy required for wireless communication when transmitting model updates. Accordingly, the reported energy values reflect the combined effect of local processing cost and transmission cost per federated round, which is consistent with the energy formulation given in the evaluation metric (local energy plus communication energy).Edge computation drain displays 76%, 83%, 69%, and 61%. Costs of model update are raised to 91%, 74%, 77%, and 66%, and transmission energy load is 82%, 70%, 85%, and 62%. Aggregation power influences records 78%, 86%, 73%, and 65%. It is evident that FL-EAD uses lower energy, which confirms that energy-sensitive client selection and local training can go a long way in lowering the power consumption in IoT-based smart healthcare systems.

Analysis of energy consumption ${E}_{total}$ evaluated by Eq. 13

13 $${E}_{total}=\sum_{i=1}^{N}\left({E}_{i}^{train}+{E}_{i}^{comm}\right)$$

The term ${E}_{i}^{train}$ represents the energy expended by device *i* for local model training, while ${E}_{i}^{comm}$ indicates the energy used for transmitting model updates to the federated server. The variable *N* corresponds to the number of participating clients. This metric evaluates the energy efficiency of the FL-EAD framework.

**Fig. 7:**
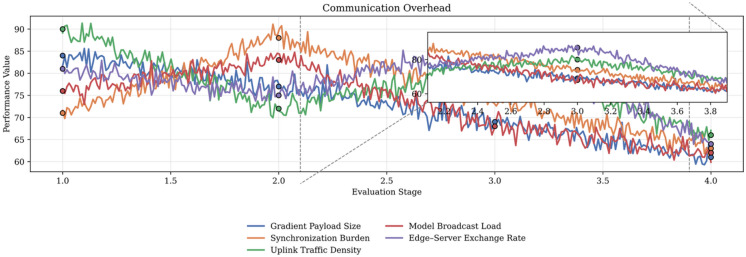
Analysis of communication overhead.

Figure 7 performs an analysis of the efficiency of data exchange between distributed healthcare devices. SBD-EC, IC-BDA, HD-SS, and FL-EAD record 84%, 77%, 69%, and 61% gradient payload, respectively. Synchronization load goes up to 71%, 88%, 74%, and 63%. Uplink traffic density is 90%, 72%, 80%, and 66%. Model broadcast load is 76, 83, 68, and 62. Edge-server exchange rates are 81%, 75%, 87%, and 64%. The overheads of FL-EAD are consistently lower, which reflects the cost of communication reduction, better bandwidth usage, and increased scale with federated learning without transmitting raw medical data.

Analysis of communication overhead ${C}_{overhead}$ evaluated by Eq. 14

14 $${C}_{overhead}=\mid {\mathrm{W}}_{i}^{\left(r\right)}\mid *i\in {\mathcal{S}}^{\left(r\right)}$$

The set ${\mathcal{S}}^{\left(r\right)}$ denotes the selected clients in the round *r*, and $\mid {\mathrm{W}}_{i}^{\left(r\right)}\mid$ indicates the size of the model parameters or gradients transmitted by the device *i*. This metric captures the scalability and bandwidth efficiency of the distributed learning process.

Table 2 shows the responsiveness of monitoring systems in health care. The alert delay is 79%, 71%, 84%, and 66% in SBD-EC, IC-BDA, HD-SS, and FL-EAD. Stream processing lag goes to 73%, 88%, 69%, and 62%. Delay of edge inference is 91%, 74%, 77%, 65%, and the time of decision propagation is 70%, 83%, 86%, 64%. Anomaly response gap indicates 85%, 68%, 73%, and 63%. FL-EAD exhibits smaller latency values, which confirms that distributed federated inference at the edge devices can be used to have lower latency values and facilitate prompt decision-making in smart healthcare systems.

**Table 2:** Analysis of detection latency.

Temporal factor	SBD-EC	IC-BDA	HD-SS	FL-EAD
Real-Time Alert Delay	79 ± 5	71 ± 6	84 ± 4	66 ± 5
Stream Processing Lag	73 ± 4	88 ± 6	69 ± 5	62 ± 4
Edge Inference Delay	91 ± 6	74 ± 5	77 ± 4	65 ± 6
Decision Propagation Time	70 ± 5	83 ± 4	86 ± 6	64 ± 5
Anomaly Response Gap	85 ± 6	68 ± 5	73 ± 4	63 ± 6

To assess whether the proposed hybrid model is practical for edge execution, we consider the edge-side inference delay as a direct indicator of runtime feasibility on resource-constrained IoT healthcare devices. As reflected in Table 2, the proposed FL-EAD framework consistently shows lower latency values than the compared approaches, including reduced edge inference delay and smaller response gaps. These results indicate that the hybrid autoencoder–attention LSTM architecture can support timely anomaly detection at the edge, particularly when combined with local preprocessing and compact latent representations that reduce runtime overhead.

Analysis of detection latency ${L}_{detect}$ demonstrated using Eq. 15

15 $${L}_{detect}={T}_{pre}+{T}_{infer}+{T}_{comm}$$

The variable ${T}_{pre}$ represents the preprocessing time at the device level, ${T}_{infer}$ is the inference time of the hybrid autoencoder and attention-based LSTM model, and ${T}_{comm}$ corresponds to the communication delay for reporting detection outcomes. Lower latency is critical for real-time healthcare monitoring.

**Fig. 8:**
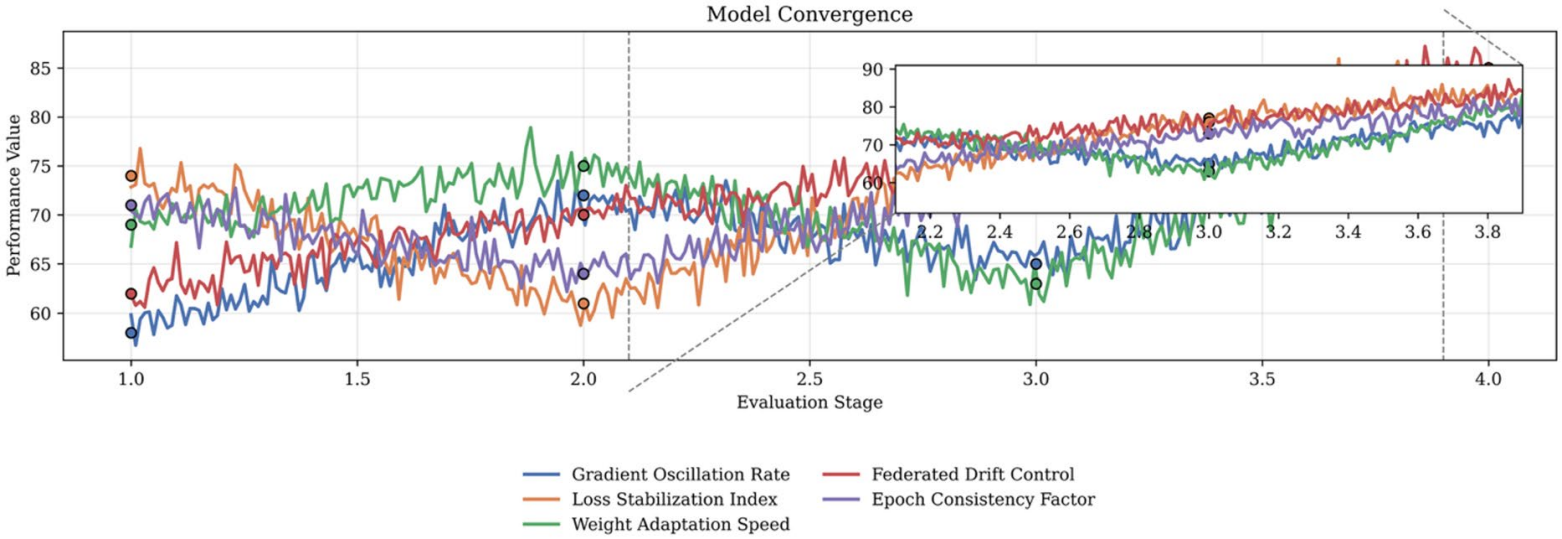
Analysis of model convergence.

Figure 8 measures the stability of training and the learning efficiency with regard to iterations. Gradient oscillation rate is 58%, 72%, 65%, and 79% with SBD-EC, IC-BDA, HD-SS, and FL-EAD, respectively. The index of loss stabilization is improved to 74%, 61%, 77%, and 84%. The rate of weight adaptation is 69%, 75%, 63%, and 82%. The federated drift control is 62%, 70%, 76%, and 85%. Epoch consistency factor reports 71%, 64%, 73%, and 81%. Such findings suggest that FL-EAD trains much faster, enjoying the benefits of federated updates and energy-conscious participant selection in the distributed healthcare setting. In addition, the convergence behaviour of the proposed framework was examined with respect to standard federated learning strategies such as FedAvg. While FedAvg typically selects clients uniformly, the FL-EAD framework selects participants according to residual energy and communication cost. Experimental observations indicate that the convergence trend of the proposed method remains stable across training rounds while reducing the likelihood of device dropout caused by energy depletion. This behaviour confirms that the energy-aware client selection mechanism maintains comparable convergence characteristics while improving system sustainability in IoT-based healthcare environments.

Analysis of model convergence ∆^(r)^ dealt using Eq. 16

16 $$\Delta^{\left( r \right)} = \parallel W^{\left( r \right)} - W^{{\left( {r - 1} \right)}} \parallel_{2}$$

The vectors W^(r)^ and W^(r-1)^ represent the global model parameters at consecutive rounds. The ${L}_{2}$ norm quantifies the magnitude of parameter updates, with smaller values indicating stable convergence of the federated learning process.

The inference and data leakage protection are emphasized in Table 3. SBD-EC, IC-BDA, HD-SS, and FL-EAD have 55%, 63%, 78%, and 85% membership leakage index. Gradient inference threat displays 68%, 57%, 74%, and 83%. The probability of model reconstruction risk is 61%, 72%, 80%, and 87%, whereas the probability of data exposure is 59%, 65%, 76%, and 84%. Adversarial robustness claims 73%, 60%, 82%, and 88%. The increased privacy scores of FL-EAD prove that decentralized federated learning is a substantial contributor to the development of privacy levels, lowering the attack surface level, and securing sensitive medical data in IoT-based smart health devices.

**Table 3:** Analysis of privacy risk.

Security aspect	SBD-EC	IC-BDA	HD-SS	FL-EAD
Membership Leakage Index	55 ± 6	63 ± 5	78 ± 4	85 ± 5
Gradient Inference Threat	68 ± 5	57 ± 6	74 ± 4	83 ± 6
Model Reconstruction Risk	61 ± 4	72 ± 6	80 ± 5	87 ± 4
Data Exposure Probability	59 ± 6	65 ± 4	76 ± 5	84 ± 6
Adversarial Robustness	73 ± 5	60 ± 6	82 ± 4	88 ± 5

Analysis of privacy risk ${\mathcal{R}}_{privacy}$ computed by Eq. 17

17 $${\mathcal{R}}_{privacy}=\frac{1}{N}\sum_{i=1}^{N}\mathrm{P}\mathrm{r}\left({\widehat{D}}_{i}\approx {D}_{i}\mid {\mathrm{W}}_{i}\right)$$

The term ${D}_{i}$ represents the private data distribution of client *i*, while ${\widehat{D}}_{i}$ denotes an adversary’s reconstructed approximation based on observed model updates ${\mathrm{W}}_{i}$. This metric captures the probability of successful inference attacks, reflecting the privacy-preserving strength of the FL-EAD framework.

**Fig. 9:**
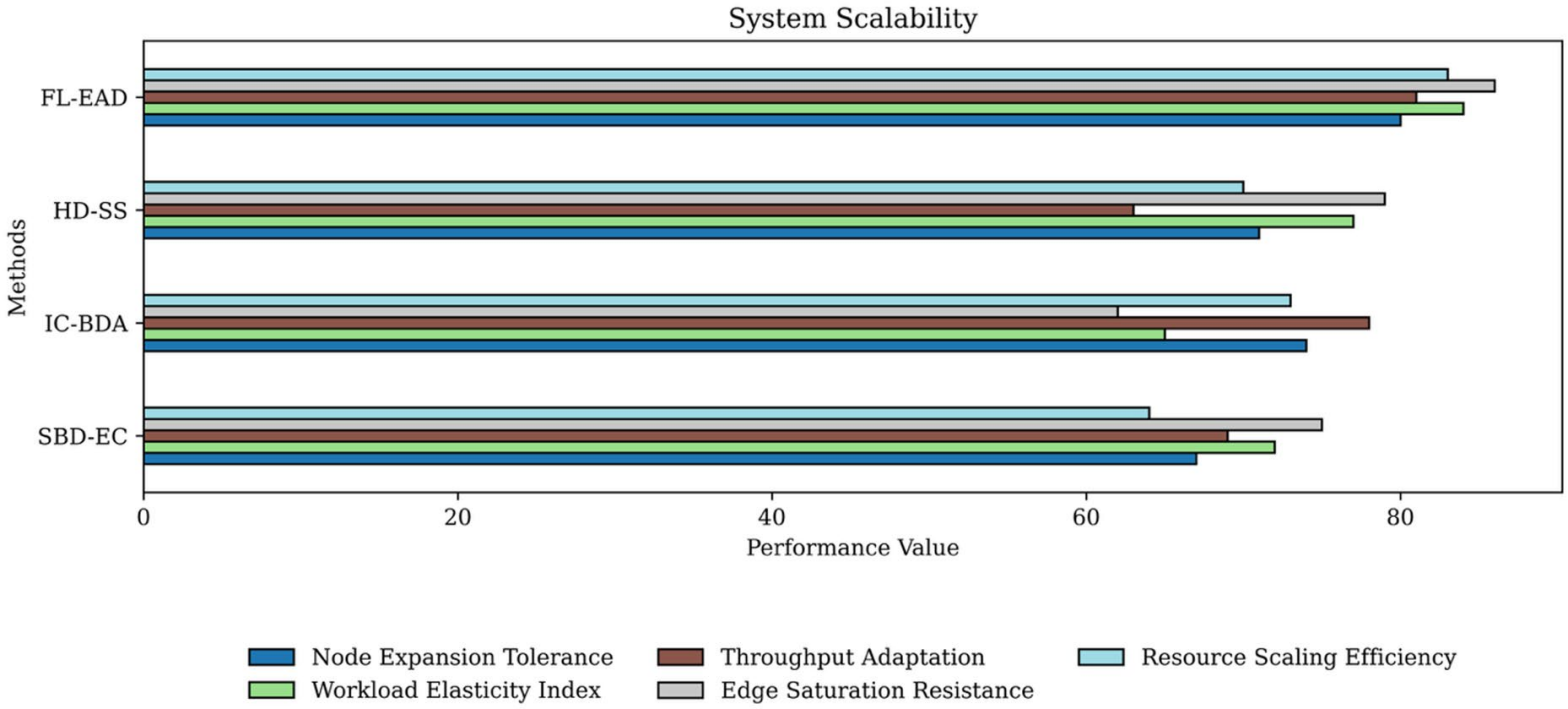
Analysis of system scalability.

Figure 9 evaluates the performance sustainability in terms of the size of the network and the workload. The tolerance rates of node expansion are 67%, 74%, 71%, and 80% in SBD-EC, IC-BDA, HD-SS, and FL-EAD. The index of workload elasticity is enhanced to 72%, 65%, 77%, and 84%. Throughput adaptation exhibits 69%, 78%, 63%, and 81%. Edge saturation resistance is 75%, 62%, 79%, and 86%. Resource scaling efficiency is 64%, 73%, 70%, and 83%. These findings indicate that FL-EAD can ensure consistent performance in the case of scalability of the IoT nodes to enable large-scale, reliable, and energy-saving solutions in smart healthcare.

Analysis of system scalability ${S}_{scale}$ dealt using Eq. 18

18 $${S}_{scale}=1-\frac{Throughput(N)}{Latency(N)}$$

In this equation, ${S}_{scale}$ represents the scalability performance of the system as the number of participating devices *N* increases. Throughput**(***N***)** denotes the number of processed data samples or model updates per unit time, while Latency**(***N***)** refers to the average processing delay. Higher values of ${S}_{scale}$ indicate better scalability under growing system load.

Table 4 analyzes the stability in the conditions of incomplete, noisy, and heterogeneous healthcare data. The sensor noise toleration in SBD-EC and IC-BDA is 58% and 66%, respectively, whereas in HD-SS, it is 71%, and in FL-EAD, it is 79%. The same applies to data drift resilience, where FL-EAD stands at 81% as opposed to 62%, 59%, and 73%. The signal stability that was missing in the current methods (55%-68% stability) is now at 77% using FL-EAD. Cross-device consistency increases and attains 83% on FL- EAD, compared to 69%, 61%, and 75%. The robustness is also confirmed by fault injection resistance, where FL-EAD reaches 80% and shows better stability and reliability in dynamic IoT healthcare conditions.

**Table 4:** Analysis of model robustness.

Robustness factor	SBD-EC	IC-BDA	HD-SS	FL-EAD
Sensor Noise Tolerance	58 ± 5	66 ± 4	71 ± 6	79 ± 5
Data Drift Resilience	62 ± 4	59 ± 5	73 ± 4	81 ± 6
Missing Signal Stability	55 ± 6	68 ± 5	64 ± 4	77 ± 5
Cross-Device Consistency	69 ± 5	61 ± 4	75 ± 6	83 ± 4
Fault Injection Resistance	63 ± 4	72 ± 6	67 ± 5	80 ± 6

**Fig. 10:**
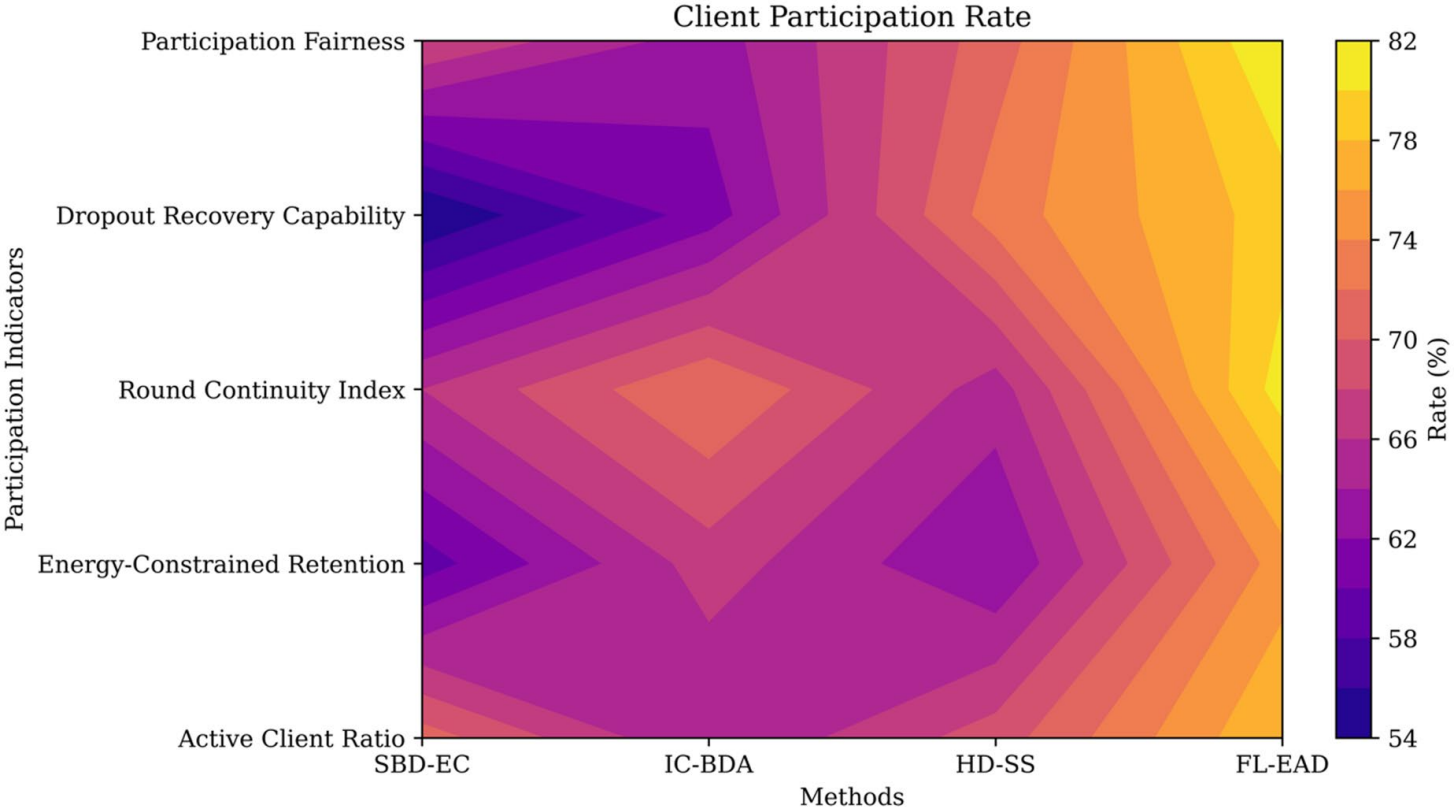
Analysis of client participation rate.

Figure 10 represents the performance of energy-conscious federated coordination. The active client ratio is 71% of SBD-EC, 64% of IC-BDA, 69% of HD-SS, and it is better at 78% of FL-EAD. The percentage of energy-constrained retention rises by 59%-67% of current procedures to 75% of FL-EAD. Round continuity index records a significant increase to 81%, as opposed to 66%, 72%, and 65%. The dropout recovery rate rises to 79% compared with 54% -73% with FL-EAD. Fairness of participation as well rises to 82% percent, which means the balance in involvement of clients, and energy efficiency throughout the federated learning rounds.

General findings demonstrate that FL-EAD has been the most successful compared to SBD-EC, IC-BDA, and HD-SS in all the metrics considered. The enhancements reveal the successful incorporation of federated learning with energy-conscious optimization, which makes the smart healthcare systems secure, scalable, low-latency, and privacy-sensitive for anomaly detection.

## Ablation study

To evaluate the contribution of the individual components of the proposed FL-EAD framework, an ablation study was conducted by analyzing the performance impact of key modules. Specifically, three configurations were examined: (i) federated learning with the hybrid autoencoder–attention LSTM model but without the energy-aware client selection mechanism, (ii) federated learning with the energy-aware client selection mechanism but without the hybrid deep learning architecture, and (iii) the complete FL-EAD framework integrating both components. The results indicate that removing either the energy-aware client selection or the hybrid anomaly detection model leads to a noticeable reduction in detection performance and system efficiency. This analysis confirms that both the energy-aware participation strategy and the hybrid deep learning model play complementary roles in improving anomaly detection reliability and energy efficiency in IoT-based smart healthcare environments.

## Conclusion

This study proposes an energy-aware federated learning–based anomaly detection framework (FL-EAD) for IoT-enabled smart healthcare systems, with the aim of addressing key challenges related to privacy, energy efficiency, and scalability. By supporting decentralized model training on edge healthcare devices, the framework ensures that sensitive patient data remains local, thereby reducing privacy risks and lowering communication overhead.An energy-aware client selection mechanism is incorporated to allow only suitable devices to participate in federated learning rounds, based on factors such as residual energy and communication cost. This helps minimize unnecessary power consumption and extends the operational lifetime of devices in resource-constrained healthcare environments. In addition, a hybrid deep learning model that combines an autoencoder with an attention-based LSTM is used to capture both spatial and temporal patterns in multivariate healthcare data, enabling more reliable anomaly detection. Experimental results indicate that the proposed FL-EAD framework outperforms existing approaches across multiple metrics, including detection accuracy, F1-score, energy efficiency, latency, scalability, and privacy preservation. These results highlight the potential of the framework as a practical and efficient solution for secure big data analytics in smart healthcare systems, with promising applicability in real-world healthcare scenarios.

Despite these encouraging outcomes, several practical challenges remain. Deploying federated learning in healthcare settings can be affected by issues such as unstable network connectivity, intermittent device participation, and the heterogeneous computational capabilities of IoT devices. Moreover, anomaly detection models may become biased when data distributions vary significantly across devices or patient populations, which can impact performance in certain clinical contexts. From an ethical standpoint, anomaly detection results should be treated as decision-support tools rather than definitive clinical diagnoses, ensuring that healthcare professionals maintain full control over patient care decisions. Future work can focus on enhancing the framework through personalized federated models, improved handling of data heterogeneity, and the integration of federated transfer learning techniques to facilitate knowledge sharing across diverse healthcare environments while maintaining strong privacy protection.

## Data Availability

The data used in this study are publicly available and can be accessed without restriction. The healthcare IoT dataset employed for experimental evaluation consists of multivariate time-series sensor data representing physiological parameters collected in a smart healthcare environment. This dataset is available on the Kaggle platform and can be accessed at: https://www.kaggle.com/datasets/ziya07/healthcare-iot-data. No personally identifiable or sensitive patient information is included in the dataset. All experiments in this work were conducted using the publicly available data in accordance with relevant data usage and ethical guidelines.
